# Supplementary material for: Correction: What matters most for early childhood development? Evidence from Malda district, India
Source: PLoS One. 2024 Jun 20;19(6):e0306034. doi: 10.1371/journal.pone.0306034 (PMC11189209; doi:10.1371/journal.pone.0306034)
Supplement: S2 File — (PDF) [file pone.0306034.s002.pdf]

PLOS ONE

RESEARCH ARTICLE

What matters most for early childhood development? Evidence from Malda district, India

Rayhan Sk *

Centre for the Study of Regional Development, School of Social Sciences, Jawaharlal Nehru University, New Delhi, India

* rayhangog@gmail.com

Abstract

Background

The early period of a child's life is considered to be the most important developmental stage throughout the lifespan. Around 250 million children of age below five years in low-and middle- income countries (LMICs) are at risk of not attaining their developmental potential. A cross-sectional case study was conducted to assess the early childhood developmental (ECD) status and to investigate the factors influencing the same in Malda, India.

Methods

Information on 731 pre-school children aged 36 to 59 months was collected in 2018 using a structured questionnaire following a multi-stage, stratified simple random sampling procedure. ECD was measured following the UNICEF's Multiple Indicator Cluster Survey, which monitors early child development in LMICs. A path analysis using structural equation modelling was carried out to examine the relationship between possible associated factors and ECD status.

Results

It has been found that only about 25% of children are developmentally on track of literacy-numeracy domain of ECD. Although, in the other domains, the situation is better. Results of the path analysis revealed that there are certain proximal factors, i.e., home environment, mother's time for care, having home tuition and attending private pre-school, which are influenced by certain distal factors and subsequently affecting ECD. The total effects revealed that place of residence (urban or rural) affects ECD mostly (Coef. -0.87, p = <0.001) directly or via mothers' time for stimulating activities, home environment and having home tuition for children. Further, it revealed that private pre-schools (Coef. 0.76, p = <0.001) and home tuition (Coef. 0.43, p = <0.001) also play a significant role in ECD.

Check for updates

OPEN ACCESS

Citation: Sk R (2022) What matters most for early childhood development? Evidence from Malda district, India. PLoS ONE 17(6): e0268985. https://doi.org/10.1371/journal.pone.0268985

Editor: Farzana Afridi, Indian Statistical Institute, INDIA

Received: January 27, 2021

Accepted: May 12, 2022

Published: June 3, 2022

Copyright: © 2022 Rayhan Sk. This is an open access article distributed under the terms of the Creative Commons Attribution License, which permits unrestricted use, distribution, and reproduction in any medium, provided the original author and source are credited.

Data Availability Statement: All relevant data are within the manuscript and its Supporting information files.

Funding: The authors received no specific funding for this work.

Competing interests: The authors have declared that no competing interests exist.

Conclusion

Urgent attention from policymakers or other stakeholders is needed to promote ECD for the betterment of children and society of Malda district. In addition to that, special attention needs to be given to the children who belong to Muslim families, socially deprived communities and living in rural areas.

1. Introduction

Early childhood development (ECD) prepares the foundation for school readiness, educational attainment, adult health and well-being, results in the formulation of social capital, ultimately leading to a prosperous and sustainable society [1–3]. A resilient foundation also lays the base for responsible citizenship, healthy communities, economic prosperity and effective parenting for the next generation. On the other hand, a fragile foundation can utterly weaken the social and economic strength of a country [4]. The early period of a child's life is considered to be the most important developmental stage throughout the lifespan [5]. Healthy early child development includes the physical, social/emotional, and language/cognitive domains of development, each equally important; that strongly influences well-being, prevents obesity/stunting, improves mental health, controls heart disease, increases competence in literacy and numeracy, prevents criminality, and enhances economic participation throughout life; all issues that have profound implications for economic burden or otherwise of countries [5]. Around 250 million children of age below five years in low-and middle- income countries (LMICs) are at risk of not attaining their developmental potential [6]. Another estimation has shown that one in three children in LMICs fails to meet basic milestones in their cognitive or socio-emotional development [7]. According to the Sustainable Development Goals (SDGs) and Global Strategy for Women's Children's and Adolescents' Health 2016–2030, every child should survive and thrive to reach their full developmental potential in their early years of life [8, 9]. A child's delayed development is mainly attributed by nutritional deficiencies, inadequate stimulation, poor environmental conditions and social risks, which are usually associated with living in poverty in LMICs [10–14]. Developmental delay adversely affects educational achievement and productivity, that ultimately leads to the intergenerational transmission of poverty and impaired development [15].

However, research on ECD in India is less known despite the fact that 10.7% of the population (110.5 million) out of a total population size of 1.21 billion were children of age 0–4 years as per the Census 2011, India [16]. One of the major reasons is that there is no national-level or secondary data source on a holistic approach of childhood development in India. Only a few surveys/studies are at the regional level in India, which were carried out by Non-Governmental Organizations (NGOs) or foreign agencies or by independent researchers, but these are not sufficient. In India, there are two major national representative surveys, National Family and Health Survey (NFHS) and District Level Health Survey (DLHS), which provide information on child health and nutrition, but they do not collect information on childhood development. Thus, it is necessary to study the status of ECD in India. Therefore, an attempt has been made in this study to conduct a cross-sectional case study at a micro level to assess the early childhood developmental status in a holistic manner, and to investigate the factors influencing the same in Malda district that is one of the bigger districts of India. The findings of this study may help policymakers to adopt appropriate interventions for a better future for the children and their families in Malda. Also, this study may add contextual insights in the

literature on early childhood development. According to the 2011 Census of India [17], the district had a total population of 3,988,845 that was almost equal to the national population of Georgia or the Oregon State of USA; also, it had a higher population than 100 countries among 233 countries which were listed in the United Nation, 2017 [18]. Further, Malda ranked 58th in terms of the size of the population out of 640 districts in India, 2011.

1.1. Factors related to child development

Children are enveloped by a hierarchical, complex, diverse social and physical environment. The proximal environment is the lowest level of the hierarchy, i.e., family settings, nested within a higher order of hierarchy or more distal environment, which all shape how children develop [19, 20]. The pioneer psychologist, Bronfenbrenner had argued in his model 'Ecological Models of Human Development' that development is related to the whole ecological system where growth takes place. The ecological system comprises five socially organised subsystems, ranging from the microsystem that includes the relationship between a developing person and the immediate environment, e.g., parents, siblings, family and school, to the macrosystem that includes forms of culture, beliefs, customs, social norms, life-styles, hazards of a particular society at a broader scale [21]. Development is a combined function of process, person, context, and time defined by the bioecological theory [22]. A very recent bio-developmental framework also emphasises the importance of children's early "nurturing, contingent, stable, and predictable" environment [23] that promotes healthy physical and cognitive development of children, especially during early years of age. The above theories suggest that there are various factors that affect ECD directly or indirectly. A large number of previous studies on the context of child development have examined the effects of various factors based on the above theories [24–27]. A review study based in developing countries has identified the four main key risk factors of child development, i.e., inadequate cognitive stimulation, stunting, iodine inadequacy and iron deficit anaemia [28]. Based on theories and empirical studies, it is found that the home environment created by the parents or the type of parenting practice is the key factor of ECD among various important factors. The ways in which children receive parental care or the home environment is also determined by multiple factors [29–33]; for example, characteristics of an individual child (age, sex, etc.); characteristics of parents (education, occupation, etc.); characteristics of households (income, caste, religion etc.); and the broader social environment where the families or households are nested (political willingness, policy programmes, level of economic development, etc.). With respect to the theories mentioned above, this study has reviewed the existing literature on the factors influencing child development thematically and mainly focused on the home environment, child's characteristics, parental characteristics and household's characteristics.

1.2. Home environment

Originally the idea of home environment came from the Home Observation for Measurement of the Environment (HOME). HOME is an instrument first developed by Bettye Caldwell and her colleagues, and they used this in a longitudinal study in 1960 to assess the quality of home environment and examined the association between home environment and childhood development [34]. The HOME inventory has been widely applied in different social and cultural backgrounds to assess the quality of home environment and its impact on cognitive and emotional development during the first five years of life. Many theorists and researchers had recognised that home environment has an independent and significant effect on childhood development [25, 34–40]. The quality of parental care or home environment has been found to be higher for children whose mothers are higher educated and for children who belong to

high-income households [30, 41–43]. Past studies have shown that mothers' interactions are usually different with their daughters and sons [44, 45]. Although, this instrument has been used widely in western countries, however, UNICEF has developed its own instrument for developing countries called 'quality of care' to assess the ideal environment which comprises a non-toxic and well-organised physical setting, opportunities for children to play, explore and discover, and the availability of books and toys, and developmentally appropriate objects in child's home environment [46]. This instrument has been used in several studies as home environment or parenting practice or parental stimulation in developing countries, and they have shown that it has a significant effect on ECD [27, 47–50].

1.3. Individual child's characteristics

Each child has his/her own specific characteristics and, the level and type of parenting given by the parents or caregivers vary by these characteristics. For example, the age of the child is one of the main characteristics, which determine the caregivers' parenting types. The previous study has demonstrated that dyadic activities between children and mothers were more responsive for older children than that of the younger ones [51]. Another study has shown that maternal talk is higher for older children than younger ones [52]. Children's sex is another important factor for receiving parenting types; in other words, the level of parenting is also influenced by the parents' perception of gender. A study has shown that the father's parenting practice is more inclined towards the male child [53]. On the other hand, studies have found that a mother's parenting activity is more inclined towards the female child [33, 54]. Further, parenting practice is also controlled by birth order. In general, and even past researches have revealed that the children of the first order of birth usually receive more parental attention as well as stimulating activates that lead to relatively higher intellectual development among them than the children born after first birth [55–57]. Previous studies have also revealed that a child's age and sex influence ECD [33, 50]. Children's gestational age is also associated with childhood development. Children born before completion of 37 weeks is called preterm birth [58], and that is adversely related to the child's behavioural and cognitive development [59, 60]. Children's nutritional status also plays a critical role in childhood development. A meta-analysis based in developing countries has established that nutritional supplementation during childhood is helpful for a child's cognitive development [61]. Besides, several cross-sectional studies have also shown that the nutritional status of children is positively associated with childhood development [62–64].

1.4. Parental and household's characteristics

Family or household is the immediate environments after a child's birth, where children start to interact [65]. The immediate environment plays a crucial role in providing support for learning, stimulation and nurturance to the children [66]. The quality of these is influenced by parental level of education, parental physical and mental health, parental stress and depression, parenting styles, housing condition and household's level of income [67, 68]. The role of maternal education in parenting practice and childhood development has been well established by a large number of empirical studies in developed counties [24, 69]. However, the effect of maternal education on home environment and subsequently on ECD is little known in developing countries, particularly at the micro-level. In relation to the outcome of childhood development, the family investment model argues that parents having a higher level of education are able to invest more capital, resources and quality time for their children than those parents having a lower level of education [70, 71]. Further, studies have shown that mothers with higher education are associated with more knowledge of ECD [72], use more

intricate language and vocabulary for their kids [73], invest more in the health of their children [74], provide more children's books at home, and also expect to have higher educational achievement from their children [75]. Moreover, higher educated mothers are likely to be more interactive and responsive towards their children, also having a higher maternal education act as a protective factor for children belong to the economically weaker sections [28]. Although, there are less studies which have examined the effect of father's educational level on the behaviour of parental practice in developing countries, however, recent studies based in Bangladesh [76], Colombia [77] and Ecuador [73] have demonstrated that paternal education has a strong positive effect on paternal stimulation and child's growth and development [49].

Contemporary trends in increasing female labour force participation play an important role in parenting practice and child's social-emotional and cognitive development. From previous studies, it can be said that the relationship between maternal occupational status and parenting practice and child development is a complex one. Mothers with high profile part-time jobs are positively associated with high quality of parenting practice and better outcomes [78–81]. On the other hand, mothers with lower occupational status are associated with higher parenting stress among mothers, lower quality of the home environment, less interactions with the child, and poor outcomes among children [82, 83]. Further, there is growing evidence that children who are witnessing domestic violence are at risk of a series of psychosocial problems [84]. Many studies in western countries have established that children who are witnessing domestic violence are at risk of wide-ranging problems, e.g., social and emotional, behavioural, psychosocial and academic etc. [85–89]. Recent studies have also suggested that mothers' level of happiness and over-stress both are associated with parenting practice and children's social and emotional development [90, 91].

In addition to these, a household's economic status is also an important factor influencing home environment and child development. A recent empirical study based in the Ghanaian context has found that households' income is the stronger predictor of holistic child development among other controlled factors [92]. It is claimed that children living in poverty are at higher risk of malnutrition, with inadequacy in protein, calcium and vitamins, which are the indispensable elements for healthy physical growth and cognitive development [93]. Household's income is also related to other risk factors, e.g., parents' psychological well-being and the aptitude to be responsive to the needs of their children [30, 94]. Several studies have also found that children belonging to economically weaker households are too weak in the process of social-emotional development, cognitive development and academic achievement than children belong to the economically advanced section [41, 67, 68, 71, 95]. Place of residence, whether urban or rural, also affects parental stimulating activities and childhood development. Past studies have also shown that mothers monitor their children more [96], and the cognitive performance is better for children [97] who belong to urban areas than mothers/children residing in rural areas. Studies on parenting practice and ECD are little known in the Indian context from the religious and caste system perspective. However, a recent study has focused on the inequalities in academic achievements among children by religious and caste groups based in India and has shown that children from Dalit, Adivasi and Muslim families are lagging than children belong to the upper caste or Brahmin households [98].

1.5. Contribution of study

From the survey of cited literature on ECD, it has been seen that there are several studies on this issue, however, they do not reflect child development in a holistic manner. Moreover, there is no study focused on India. Thus, it is necessary to study ECD in a comprehensive manner in India. Most of the previous studies focused on a particular aspect of ECD. Majority of

them have focused on the cognitive development of children only and have neglected the other domains of child development. Whereas, according to UNESCO, 2014 *"Children's development is holistic*: *cognitive*, *language*, *physical and social-emotional development all work together. Progress (or lack of progress) in one domain spurs or hinders development in other domains in a dynamic process"* [99]. Thus, studies are needed to capture all the domains (cognitive, language, physical and social-emotional development) of ECD to reflect the early childhood developmental status in a holistic manner. Further, most of the previous studies tried to predict the direct effects of socio-economic status (SES) or child characteristics which act as proximal factors of children's cognitive development or either study focused on effects of parental practices or home environment on children's cognitive development exclusively. Since the indirect effects of these factors are little known; therefore, studies are needed to examine the indirect effects. Furthermore, the relationship between children attending pre-school or private pre-school and child development has not been explored much. The association between children having home tuition and child development also needs to be investigated. Also, the time given by the parents for stimulating activities has not been examined in the case of child development. Therefore, taking into account all above these limitations in the existing studies, an attempt has been made in this study to conceptualise a framework (Fig 4) and to conduct a cross-sectional survey at the regional level to assess ECD status in a holistic manner among pre-school children aged 36–59 months, and to identify factors influencing the ECD. Further, based on the previous studies, the present study assumes that creating a home environment where children are being reared after his or her birth is an art of the mother or parent, which is being influenced by the parent's socio-economic characteristics and child characteristics. It has been assumed that differences in child development depend on the quality of the home environment or parenting practice largely.

2. Methods

2.1. Sampling and data

A primary cross-sectional sample survey was conducted to represent the children (36–59 months) of the Malda district of West Bengal, India, in 2018. It was designed to collect the information of pre-school children aged 36 to 59 months, especially to collect the ECD indicators and additionally, the indicators of quality of care or home environment, parent's characteristics, and household's characteristics were also collected for rural and urban areas from the 12 selected primary sampling units (PSUs) which were nested within the three selected blocks and a municipality. The villages in rural areas and wards in urban areas were considered as the PSUs. Generally, a sub-district, i.e., Tehsil or Taluk or block, is the administrative unit under a district in rural India. However, there are some exceptions in some states and union territories since the classification for sub-districts is not identical across the country. On the other hand, a municipality is an urban local body in India. Ethical approval was obtained from the IERB-JNU (Institutional Ethics Review Board Jawaharlal Nehru University), New Delhi, IERB Ref. No.2019/Student/223. The purpose of the study was thoroughly explained to respondents, and written consents were obtained before starting the survey.

A multi-stage, stratified simple random sampling procedure was used to collect the survey sample. Considering the researcher's time, affordability and feasibility, a sum of 750 samples were expected to be collected. The Census 2011, India, was used for framing the sampling procedure. In the beginning, the district was stratified by the rural and urban population, and the sample was distributed according to the proportion to population size. According to the Census 2011, 13.6% and 86.4% of the population were urban and rural residents, respectively. Thus, the total expected sample for the urban and rural population was 102 (750/100*13.6)

and 648 (750/100*86.4), respectively [17]. There were 15 Blocks or Tehsils, the higher-level administrative units in rural areas in Malda district. These were stratified into three groups using the tertile method after ranking them by z-scores of relative wealth index that was a composite measure, constructed with certain variables like household's assets, amenities and facilities using data from the Census 2011, India [17]. Afterwards, one block had been selected randomly from each group in the first stage of sampling. The tertile method for stratification was also used to represent the children from all the sections of Malda district. A total of three blocks had been selected, and the sample size had been distributed according to the proportion to population size. On the other hand, in the case of urban areas, a municipality had been selected randomly from the two municipalities, and all the urban samples have been taken from that selected municipality.

In the second stage of sampling, PSUs or villages/wards had been selected. Likewise blocks stratification, villages/wards were also stratified into three groups following the same process. After this, one village/ward had been selected randomly from each group of villages/wards. Thus, three PSUs had been selected from each selected block or municipality. A sum of 12 PSUs, nine PSUs/villages from rural areas and three PSUs/wards from urban areas had been selected. Here, at the village/ward level, the samples had been fixed equally by dividing the total expected sample by the total selected villages/wards for the corresponding block or municipality. Since the present research was designed to study the ECD status of children of aged 36 to 59 months, therefore, only households that had at least one child of age ranging between 36 to 59 months were included in the present study. The listing of households with at least an eligible child had been made from various sources in the selected PSUs. In the final stage, after completion of house listing, the desired number of households for the corresponding PSU had been selected using a simple random sampling procedure.

After selecting the households with at least one child aged between 36 to 59 months, there were two major conditions that had been followed for selecting a child with the mother or caregiver who was the primary respondent of the survey questionnaire. These were, i) child must be between 36 to 59 months of old at the time of the survey; ii) if there were more than one child aged between 36 to 59 months in a selected household, thus only one child had been selected following the alphabetic order of child's name, and the survey questions had been asked to the mother or caregiver of that particular selected child. The expected sample size was 750. However, a sum of 731 mothers or caregivers had responded and completed the survey, and the remaining 19 sample were not interviewed due to either unavailability of the respondents or their non-response to the survey. Thus, the overall survey response rate was 97.5%. The response rate for urban areas was 98%, while the response rate for rural areas was 97.4%.

2.2. Estimation procedure

Outcome variable. To measure the early childhood developmental status, this study has followed the Multiple Indicator Cluster Survey (MICS, 4th round), which monitors early child development in low and middle-income countries, conducted by UNICEF [46]. The MICS is one of the only global sources to measure children's outcomes in a holistic manner in the early childhood years on the basis of mother's/caregiver's observations. A 10-item module developed by the UNICEF has been used to measure the early child development status in this study, and this measure has also been used in other studies [7, 48, 50, 100]. This measure is based on selected milestones that children are expected to achieve by the ages of three and four [46]. However, one more item has been added for this study. Thus, a total of 11 items have been taken into consideration for this study and asked to the caregivers or mothers during the survey to assess the ECD status. These are included in four main domains as follows:

(*1*). *Literacy–numeracy*: Children have been considered to be developmentally on track in this domain if any two of the followings four (i-iv) conditions are fulfilled; (i) they can identify/ name: a minimum of 10 letters of the alphabet, (ii) they can read a minimum of four simple, popular words, and (iii) they can recognise the name and distinguish the symbols of all numbers from 1 to 10. Besides these, one more item has been added in this research, that is (iv) whether children can count the numbers from 1 to 10. The last item added since there is a substantial variation in responses to this question in the present study setting.

(2). *Physical*: (i) If the child can pick up a small object with two fingers, like a stick or a rock from the ground and/or (ii) the mother/caretaker does not indicate that the child is sometimes too sick to play, then the child has been considered to be developmentally on track in this domain.

(3) Social-emotional: Children have been identified as being developmentally on track in this domain if at least two of the following conditions are fulfilled: (i) the child gets along well with other children; (ii) the child does not kick, bite, or hit other children; and (iii) the child does not get distracted easily.

(4). *Learning*: Children have been identified as being developmentally on track in the learning domain (i) if the child follows simple directions on how to do something correctly and/or (ii), when given something to do, is able to do it independently.

For the purpose of this study, each item in the domains of ECD has been scored in a binary fashion. A score of 1 indicates the presence of a positive aspect of ECD, and a score of 0 indicates the absence of a positive aspect of ECD. However, only eight items out of 11 items have been considered to measure the ECD status in this study. Since the response of mothers or caregivers regarding the physical domain and the response of whether a child gets along well with other children were almost universal thus, these were not taken into consideration. Therefore, this study has only focused on eight items of the remaining three domains, namely, literacy-numeracy, social-emotional and learning domains. A composite score for ECD based on the eight items was produced by summing up the number of positive responses for the literacy/numeracy, social/emotional, and learning domain items, with scores ranging from 0 to 8 [101]. The lower the score, the lower is the level of ECD status and the higher the score, the higher is the level of ECD status.

2.3. Explanatory variables

Endogenous variables. The parenting practices or home environment created by the parents was assessed through a set of questions based on UNICEF's learning materials and support for learning tools [46], which had been asked to the mothers or caregivers during the field survey. Accordingly, 'home environment' or the quality of care given by parents at home includes (i) whether the children have access to three or more children's books and (ii) manufactured toys, (iii) homemade toys, (iv) household's objects (such as sticks, rocks, shells, etc.); whether the (v) mother, (vi) father, (vii) any adult member of the household and (viii) other adult household's member are engaged at least in four of the following activities with the child, i.e., reading books, telling stories, singing songs, going outside, playing and counting, naming and drawing. A composite score for home environment based on the eight items was produced by summing up the number of positive responses for learning materials and support for learning tools, with scores ranging from 0 to 8. The lower the score, the lower is the quality of home environment; and higher the score, higher is the quality of home environment. This measure has also been used in a study [102]. The home environment has been considered as an

endogenous or proximal variable in the analysis as influenced by other distal factors in the conceptual framework (Fig 4). The other endogenous variables are whether children are attending private pre-schools (dummy: no vs. yes), whether children have home tuition (dummy: no vs. yes) and the mother's daily time allocated (approximately) for various stimulating activities with their children (continuous: in hours), which are also influenced by multiple exogenous factors. Caregivers or mothers were asked about their as well as their husbands or fathers of children on the average daily allocated time for various stimulating activities with their children during the survey.

Exogenous variables. Among the child's characteristics, child's age (continuous: in months), sex (dummy: male vs. female), birth order (continuous: low to high), premature birth (dummy: no vs. yes), stunting (dummy: no vs. yes) are considered as the exogenous or distal variables. Among the parental characteristics, mother's education (ordinal: level of educational attainment), mother's occupation (dummy: others vs. Bidi/agricultural worker), domestic violence experienced by mothers (dummy: no/don't want to say vs. yes), mothers' level of happiness (ordinal: very happy to very unhappy), fathers' education (ordinal: level of educational attainment) and fathers' time for stimulating activities (continuous: in hours) are considered as the exogenous variables. A question was asked to the mothers/caregivers on the level of happiness with a five-level order ranges from very happy to very unhappy at the time of the survey, and this variable has been treated as an ordinal variable in this analysis. Also, the level of mother and father's education have been categorised into five groups: (0) none, (1) primary, (2) lower secondary, (3) upper secondary, and (4) higher, and treated as the ordinal variables for this study [92]. A dummy variable has been created for mothers' occupation status. Agricultural workers, manual labourers and bidi workers have been referred to as 'bidi/agricultural worker' and considered as workers engaged in the low-status work, whereas the private or public service holders and housewives are referred to as 'others' and have been considered relatively high-status workers. Among household's characteristics, family type (dummy: single family vs. joint family), wealth index (continuous: scores), religion (dummy: Hindu vs. non-Hindu or Muslim/others), caste [dummy: non-SC/ST vs. SC/ST (Scheduled Caste/Scheduled Tribe)] have been taken as exogenous factors. The wealth index is a proxy measure of income, which has been created using the selected household's assets, amenities and facilities following the Demographic Health Survey (DHS) (Rutstein & Johnson, 2004). SC which traditionally had the lowest social standing, and ST, a group of tribes who were isolated. SC and ST are considered as the most deprived community in India. Among institutional factors, whether children are attending AWCs/ICDS (dummy: no vs. yes) has been considered as an independent factor in the analysis. ICDS is one of the most inclusive programmes for addressing child malnutrition and for child well-being and development. ICDS is operating through Anganwadi Centres (AWCs), which work as the first outpost for nutrition, health and early learning services at the grassroots level or the village level in rural areas and the ward level in urban areas. The AWC is operated by an Anganwadi Worker (AWW) and an Anganwadi Helper (AWH). The AWW is accountable for providing supplementary nutrition, non-formal pre-school education and health education [103–105]. Besides these, place of residence (dummy: urban vs. rural) has been included as an independent variable from community characteristics. All the first category of dummy variables has been coded as '0', and the second category has been coded as '1' in this study.

2.4. Analytical plan

Based on the existing theories of child development and survey of the literature, a diagrammatic presentation of the conceptual framework has been presented in Fig 4 in the results

section. The figure shows that child development is an outcome of certain proximal factors and a large number of distal or exogenous factors. The potential proximal or endogenous factor of child development is the home environment or parenting practice. The home environment is influenced by the child's characteristics such as the child's age, sex and birth order. In addition, the home environment is also influenced by parental characteristics such as mother's education, occupation, level of happiness, domestic violence experienced by mothers, and the father's education and by household characteristics such as level of income, religion, caste or social groups and place of residence. Other important proximal factors are whether a child is attending private pre-school, whether a child has home tuition and the mother's allocation of time for various stimulating activities for their children. These proximal factors are also controlled by certain parental factors and household characteristics, as shown in the diagram. On the other hand, the distal factors also affect ECD directly or indirectly through the proximal factors. For example, individual child characteristics affect the ECD indirectly through the quality of home environment or parental stimulating activities and also influence the level of child development directly. Parental characteristics affect child development in various ways. All parental characteristics affect child development directly. Several parental characteristics influence child development via home environment. Further, some of the parental characteristics, i.e., level of mother's and father's education, affect child development indirectly through the type of pre-schools or home tuition and home environment. Household characteristics also affect ECD in various ways. Household characteristics affect directly as well as indirectly through the home environment and by the other proximal factors that have been shown in the diagram. Further, it has been assumed that whether children are attending AWCs or other pre-schools will affect child development, as shown in Fig 4.

Descriptive statistics were computed to understand the characteristics of the study children. Child development is a complex process where predictors can play a role directly as well as indirectly through various ways. Also, the hypothesised conceptual framework involved complex path models with a number of intermediates and independent factors that affect ECD. In that case, multivariate analysis or multiple linear regression model is unable to examine the path relations or to estimate the indirect effects of various predictor variables, also unable to determine the mediating factors. So, this needs a more sophisticated and rigorous statistical technique to testing several relationships concurrently. Structural equation modelling (SEM) allows one to test complex models involving a number of intermediates and independent factors simultaneously [106]. SEM is designed to evaluate how well a conceptual model that contains observed indicators and hypothetical constructs explains or fits the collected data [107, 108]. SEM is a suitable method to measure the direct effects, indirect effects and total effects of exogenous and endogenous variables on outcome variable simultaneously [109]. Based on these observations, SEM is found to be the best-fitted model with the present study's conceptual framework. Therefore, a path analysis using SEM was carried out to examine the direct effects of selected child characteristics, parental characteristics and household characteristics on ECD and their indirect effects on it through home environment, private pre-school, home tuition, and mother's time for various stimulating activities which are acting as the mediators in the conceptual framework. Path analysis was developed to quantify the relationships among multiple variables, and that can explain the causal relationships among multiple variables [106, 110]. SEM lets us set up and test a plausible path model through which factors are linked to ECD. The "sem" command used in STATA (v. 13.1), which fits structural equation models to estimate the direct and indirect effects [109]. All variables included in the model were observed variables (none are calculated as latent). SEM allows one to produce a visual illustration of the model and to estimate a series of models to obtain direct, indirect, and total effects of exogenous variables on the outcome of interest [111]. SEM cannot demonstrate causality, and the

Table 1. Descriptive statistics of the study children (36–59 months) in Malda (n = 731), 2018.

Characteristics	*Sample mean (SD) / percentage
**Child's age** (continuous)	47.5 (6.6)
**Child's sex** (dummy)	
Male	51.6
Female	48.4
**Birth order** (continuous)	2.0 (1.1)
**Premature birth** (dummy)	
No	81.8
Yes	18.2
**Stunting** (dummy)	
No	59.9
Yes	40.1
**Mother's education** (ordinal)	1.9 (1.2)
None	18.3
Primary	15.7
Lower secondary	35.6
Higher secondary	20.1
Higher	10.3
**Mother's occupation** (dummy)	
Others	64.2
Bidi/agricultural worker	35.8
**Mother's time for stimulating activities** (continuous)	0.7 (0.7)
**Domestic violence** (dummy)	
No	87.7
Yes	12.3
**Mother's happiness** (ordinal)	2.4 (1.1)
**Father's education** (ordinal)	1.5 (1.2)
None	25.2
Primary	23.1
Lower secondary	30.8
Higher secondary	12.9
Higher	06.8
Missing	01.2
**Father's time for stimulating activities** (continuous)	0.2 (0.3)
**Family type** (dummy)	
Single	83.3
Joint	16.7
**Wealth index** (continuous)	4.8 (2.4)
**Religion** (dummy)	
Hindu	43.9
Muslim/others	56.1
**Caste** (dummy)	
Non-SC/ST	74.3
SC/ST	25.7
**Place of residence** (dummy)	
Urban	13.7
Rural	86.3
**Attending AWC** (dummy)	

(*Continued*)

Table 1. (Continued)

Characteristics	*Sample mean (SD) / percentage
Yes	60.7
No	39.3
**Attending private pre-school** (dummy)	
No	76.6
Yes	23.4
**Home tuition** (dummy)	
No	78.3
Yes	21.8

Note:
*Sample mean (SD) for continuous/ordinal variables; percentage distribution for dummy variables.
Sources: Primary field survey, Malda, 2018.

https://doi.org/10.1371/journal.pone.0268985.t001

results should be interpreted as correlations; however, it does allow one to determine whether the hypothesised causal pathway is plausible or not [112]. Maximum likelihood estimation is the default estimation method in many SEM software [113, 114], and that method has been applied for the present analysis. The direct coefficients (effects), indirect coefficients (effects) and total coefficients (effects) of exogenous and endogenous variable on ECD score have been estimated and presented in a single table (Table 2). A p value <0.05 has been considered as statistically significant. Also, 95% confidence intervals (CI) have been presented along with the p values in the table. The following formula has been used to compute the indirect effect and total effect of variable "k" on the outcome variable using "nlcom" command in Stata 2013 [109]:

$$\text{“}Indirect\ \ effect = \beta_{12}\ \ \beta_{\mathrm{k1}}$$

$$Total\ \ effect = \beta_{\mathrm{k2}} + \ \ \beta_{12}\ \ \beta_{\mathrm{k1}}$$

Where,
β_{12} = *path coeffcient from endogenous variable to* the *outcome* variable
β_{k1} = *path coeffcient from exogenous variable* k *to* the *endogenous* variable
β_{k2} = *path coeffcient from exogenous variable* k *to* the *outcome* variable

If an exogenous variable is not postulated to inference the endogenous variable, then obviously there is no indirect effect, and the total effect is simply the direct effect β_{k2}*"* [111].

3. Results

3.1. Descriptive statistics of the study children

Table 1 presents the descriptive statistics of the study children. The average age of the study children is 47.5 months with the (SD) standard deviation of 6.6. While 51.6% of children are male, and the remaining 48.4% are female. The mean birth order is two with 1.1 SD. About 18% of children were born before the completion of 37 weeks of gestational age. Almost four in 10 children are stunted. Nearly 18% of mothers did not have any formal education, 15.7% of mothers have attained primary education, 35.6% of mothers have attained lower secondary education, 20.1% of mothers have attained higher secondary education, and 10.3% of mothers

have attained a higher level of education. About 36% of mothers are bidi worker or agriculture workers. Almost 12.3% of mothers have reported that they have experienced domestic violence either by husbands or by any other household members in the household last month before the survey. In the study area, the level of the father's education is poor as compared to the mother's level of education. About 25% of fathers do not have any formal education, 23.1% of fathers have attained primary education, 30.8% of fathers have attained lower secondary education, 12.9% of fathers have attained higher secondary education, and only 6.8% of fathers have attained a higher level of education. A larger proportion of the households (83.3%) are of single family type, and the remaining 16.7% of households are of joint family type. About 44% of households are Hindus, whereas 56% of households are Muslims or other minor religious group. Almost one-fourth of children belong to socially deprived communities like scheduled tribe (ST) or scheduled cast (SC). The majority of the child population (86.3%) are living in rural areas, and only 13.7% of children are living in urban areas. A larger part of study children, about 61% of children are attending the AWCs, and the remaining 39% are not attending AWCs or attending other types of pre-schools. In contrast, about 23% of children are attending private pre-schools among the total study children. Further, about 22% of children have home tuition in the study setting.

3.2. Percentage distribution of children by indicators of ECD in Malda, 2018

Fig 1 portrays the percentage distribution of children along with the indicators of ECD based on mothers or caregivers' observations. It is observed that only 31.7% of children can identify ten letters of the alphabets among the sampled children, and 17.7% of children are able to read four simple popular words. About 40% of children can count the numbers from 1 to 10, whereas about 24% of children can recognise numbers from 1 to 10. Almost 98% of children can pick up any small object from the ground, and about 92% of children do not feel too sick to play. About 83% of children can follow simple directions on how to do something correctly, while nearly 48% of children are able to do something independently when it is given to them. About 99% of children get along well with the other children. However, 77% of children kick, bite or hit other children or adults, and the remaining proportion, i.e., 23% of children, do not kick, bite or hit others. Almost 93% of children do not get distracted easily in the study sample.

3.3. Percentage distribution of children by domains of ECD in Malda, 2018

From Fig 2, it has been seen that about 25% of children are on track in the literacy-numeracy domain of ECD as per the UNICEF's measurement tools, 2014. However, in the case of the learning domain, about 83% of children are developmentally on track in this domain. About 95% of children are developmentally on track in the domain of social and emotional development. On the other hand, almost 99% of children are physically fit, as reported by the mothers or caregivers of the children.

3.4. ECD mean score by children's background characteristics in Malda, 2018

Fig 3 presents the mean score of ECD along with error bars (computed from 95% confidence intervals) by each category of children's background characteristics. The overall mean ECD

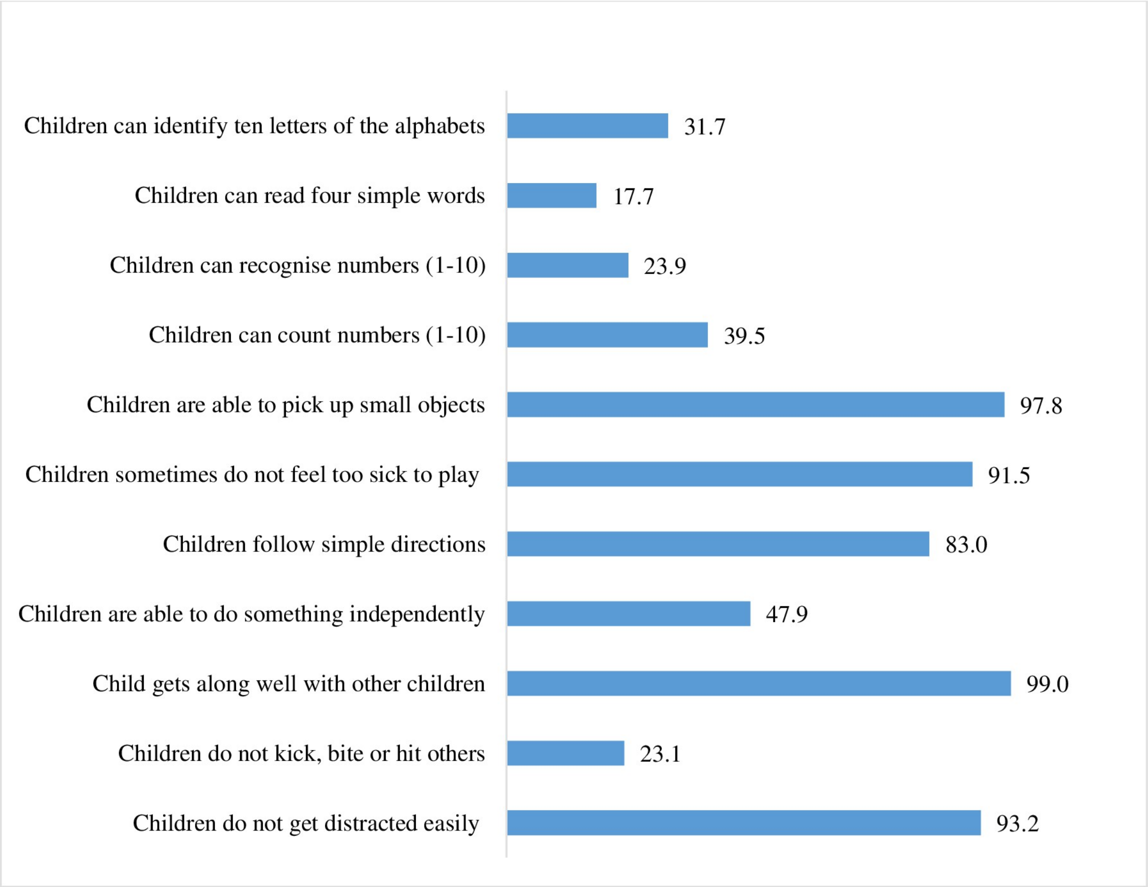

Fig 1. Percentage of children by indicators of ECD in Malda, 2018.

https://doi.org/10.1371/journal.pone.0268985.g001

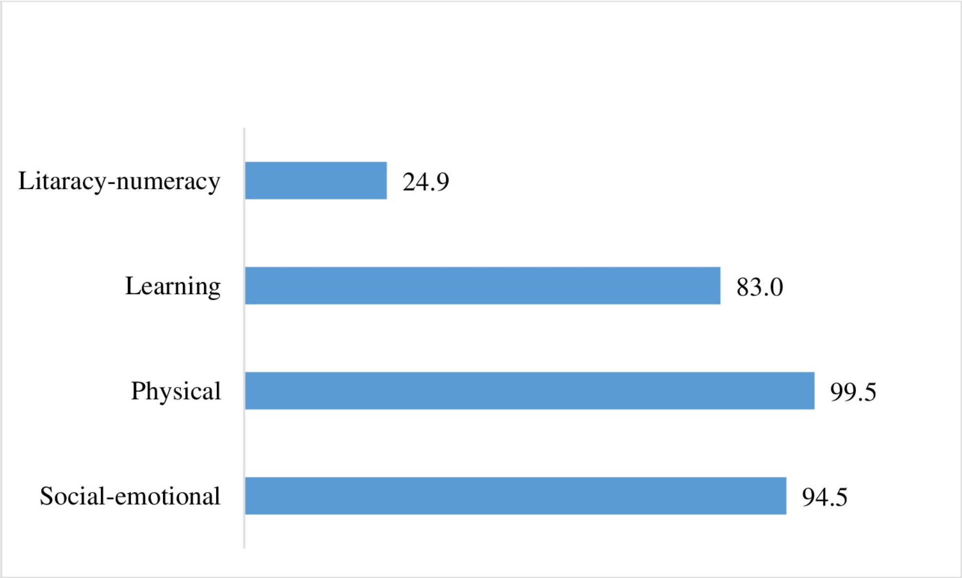

Fig 2. Percentage distribution of children by domains of ECD in Malda, 2018.

https://doi.org/10.1371/journal.pone.0268985.g002

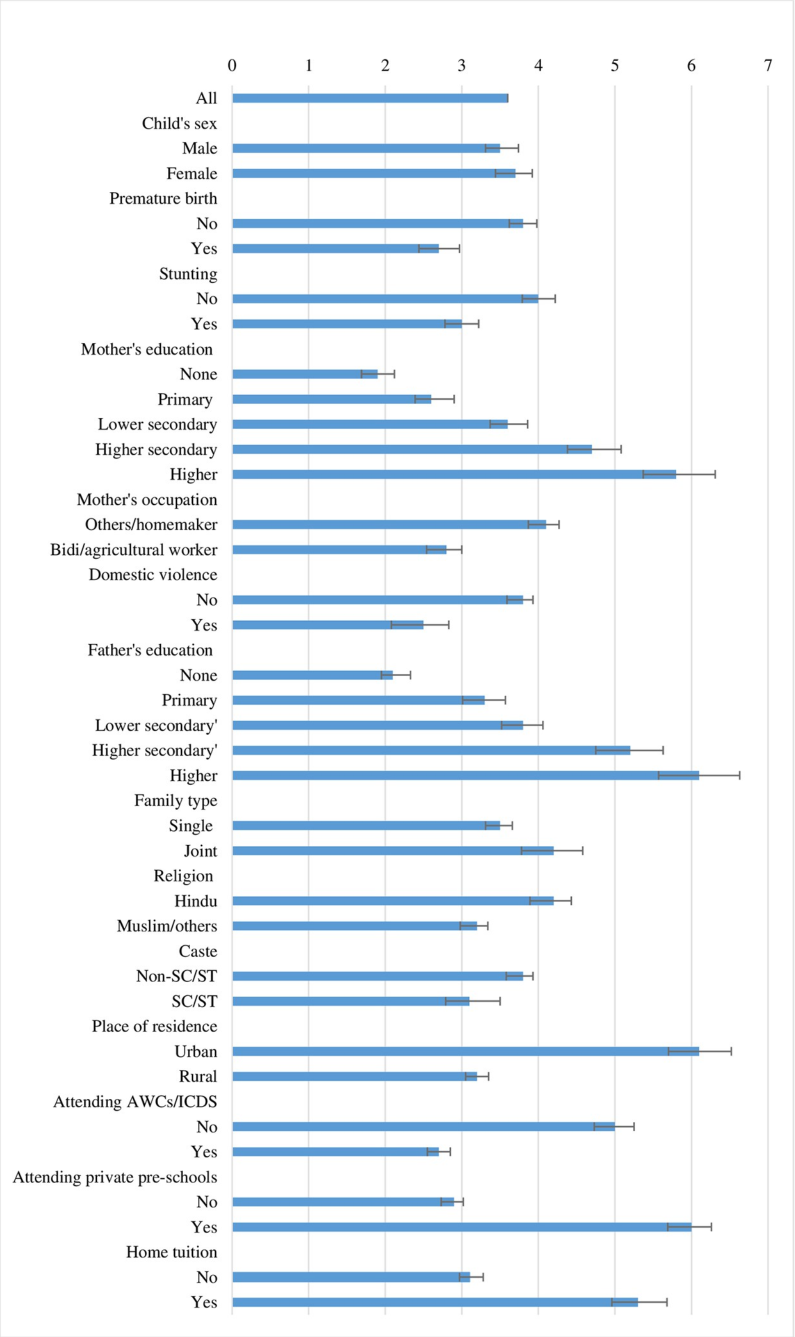

Fig 3. ECD mean score by background characteristics in Malda, 2018.

https://doi.org/10.1371/journal.pone.0268985.g003

score for the sampled children is 3.6 out of 8. The mean scores for female and male children are almost the same, though the female children are somehow doing better than the male children. About a one-point difference is observed between preterm birth and non-preterm birth. Children with premature birth are less advanced than their counterparts. Again, a one-point difference is found between stunted and non-stunted children, where non-stunted children

are in an advanced position. A considerable variation in the mean score of ECD has been observed across the level of mothers' education. This is ranging from 1.9 for illiterate mothers, 2.6 for primary education, 3.6 for lower secondary education, 4.7 for higher secondary education, and 5.8 for higher educated mothers. The mean score for Bidi/agriculture or other labourers is lower (2.8) than that of the homemakers or higher working status (4.1). The mean score of ECD is lower for children belong to mothers who have experienced any domestic violence as compared to children whose mothers have not experienced the same. A higher difference in the mean score of ECD is witnessed across the level of fathers' education as well, ranging from 2.1 for illiterate fathers, 3.3 for primary education, 3.8 for lower secondary education, 5.2 for higher secondary education, and 6.1 for higher educated fathers. The mean score of ECD is higher for children who belong to joint families in comparison to the children who belong to single families. One point of difference in mean score is observed between Hindus and Muslims, where children from Muslim or other minor religious group is lagging. Children belonging to the SC/ST households are less advanced in ECD than children belong to non-SC/ST households. The place of residence, whether rural or urban areas, makes a large difference in the mean score. The mean score of ECD for children living in urban areas is almost double (6.1) than that of the children living in rural areas (3.2). A large gap in the mean score of ECD is witnessed between the children who access AWCs and the children who do not access the same or otherwise. Children who are not attending the AWCs are in an advanced position than the children attending the same. A large gap in the mean score of ECD is also seen between the children who access private pre-schools and children who do not access the same. Children attending the private pre-schools (6.0) are in an advanced position than the children who are going to the AWCs or somewhere else or nowhere (2.9). Further, a notable gap in the mean score of ECD is also found between the children who have home tuition and children who do not have. Children who have home tuition are in an advanced position in the mean score of ECD (5.3) than the children who do not have home tuition (3.1). In a nutshell, a relatively higher level of variations in the mean score of ECD is observed in the case of the mothers' and fathers' level of education, place of residence, access to AWCs or attending private pre-schools and having home tuition or not, as observed in Fig 3.

3.5. Results of path analysis

Path analysis using structural equation modelling has been employed to examine the direct and indirect association between the explanatory variables, endogenous variables and outcome measure. The estimated path coefficients of the path models are also presented along with the conceptual framework in Fig 4. Further, direct effects or direct path coefficients, indirect effects or indirect path coefficients and total effects (direct + indirect effects) have been presented in Table 2.

The total effects of exogenous and endogenous variables reveal that several factors considered in the conceptual framework are influencing ECD. Among them, major statistically significant factors of ECD are the place of residence, type of pre-schools, home environment, home tuition, mother's education and mother's time for stimulating activities. Bedside these, other important factors are father's education, wealth index and whether children are attending AWCs or not.

The home environment created by the parents also plays a significant role in ECD. Expectedly, it is positively associated (Coef. 0.46, $p = <0.001$) with the ECD. There are several significant predictors of home environment. Among them, major factors are the place of residence, religion, mother's education and wealth index. Of them, rural areas as a place of residence and

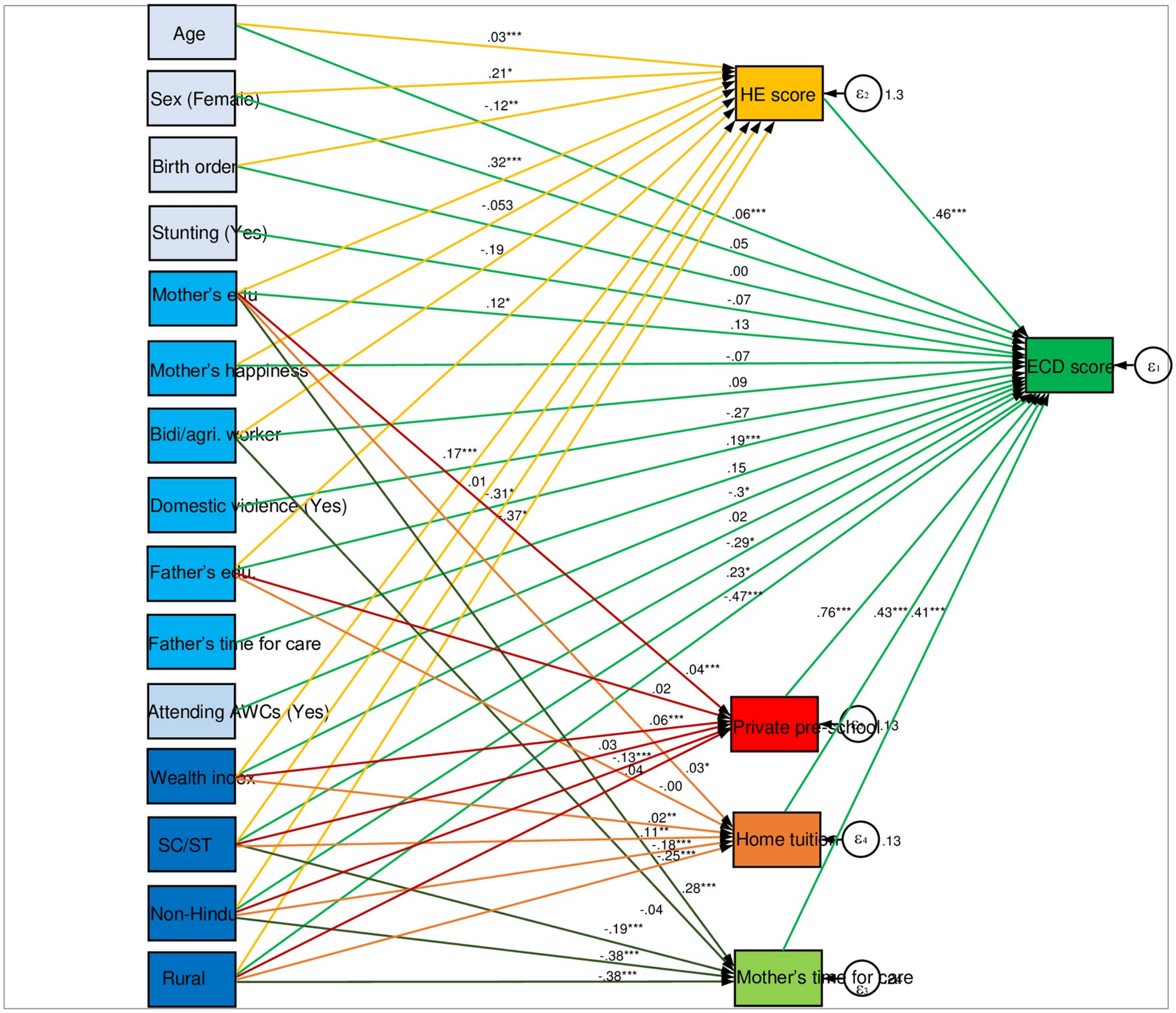

Fig 4. Conceptual framework and estimated path coefficients using structural equation modelling.

https://doi.org/10.1371/journal.pone.0268985.g004

Muslims/others as a religious group are negatively associated with home environment, while mother's education and wealth index are positively associated with the same.

Among child's characteristics, age is the only associated factor of ECD, where it is positively correlated.

Mother's educational level is also an important factor of ECD, shown by total effects. Expectedly, mother's education is playing a positive role (Coef. 0.43, p = <0.001) in the process of child development. A larger part of the effect of mother's education (Coef. 0.30, p = <0.001) is accounted by the indirect way through home environment and mother's time for stimulating activities. Father's educational level also plays an important role in ECD status, and this is also positively associated (Coef. 0.26, p = <0.001) with ECD, shown by total effects. However, the effect of the father's education is mostly reported through the direct path (Coef. 0.19, p = <0.001). The wealth index is also positively associated (Coef. 0.15, p = <0.001) with ECD, reported by total effects; though most of its effect (Coef. 0.13, p = <0.001) has been passed via

Table 2. Estimated coefficients (effects) of exogenous and endogenous variables on ECD score using SEM, Malda, 2018.

Characteristics	Direct effects	Indirect effects	Total effects
	Coef. [95% CI]	Coef. [95% CI]	Coef. [95% CI]
HE score (endogenous variable)			
Child's age	0.03***[0.02–0.04]	- -	0.03***[0.02–0.04]
Child's sex (Female)	0.21*[0.04–0.38]	- -	0.21*[0.04–0.38]
Birth order	-0.12**[-0.20–0.03]	- -	-0.12**[-0.20–0.03]
Mother's education	0.32***[0.22–0.42]	- -	0.32***[0.22–0.42]
Father's education	0.12*[0.03–0.21]	- -	0.12*[0.03–0.21]
Mother's happiness	-0.05[-0.14–0.03]	- -	-0.05[-0.14–0.03]
Wealth index	0.17***[0.11–0.23]	- -	0.17***[0.11–0.23]
Caste (SC/ST)	0.10[-0.13–0.32]	- -	0.10[-0.13–0.32]
Religion (Non-Hindu)	-0.31**[-0.51–0.11]	- -	-0.31**[-0.51–0.11]
Place of residence (Rural)	-0.37*[-0.71–0.04]	- -	-0.37*[-0.71–0.04]
Mother's occupation (Bidi/agri. worker)	-0.19[-0.39–0.01]	- -	-0.19[-0.39–0.01]
Mother's time for care (endogenous variable)			
Mother's education	0.28***[0.24–0.31]	- -	0.28***[0.24–0.31]
Caste (SC/ST)	-0.19***[-0.28–0.09]	- -	-0.19***[-0.28–0.09]
Religion (Non-Hindu)	-0.38***[-0.47–0.30]	- -	-0.38***[-0.47–0.30]
Place of residence (Rural)	-0.38***[-0.49–0.26]	- -	-0.38***[-0.49–0.26]
Mother's occupation (Bidi/agri. worker)	-0.04[-0.13–0.05]	- -	-0.04[-0.13–0.05]
Home tuition (endogenous variable)			
Mother's education	0.03*[0.00–0.06]	- -	0.03*[0.00–0.06]
Father's education	0.00[-0.03–0.03]	- -	0.00[-0.03–0.03]
Wealth index	0.02**[0.01–0.04]	- -	0.02**[0.01–0.04]
Caste (SC/ST)	0.11**[0.04–0.17]	- -	0.11**[0.04–0.17]
Religion (Non-Hindu)	-0.18***[-0.25–0.12]	- -	-0.18***[-0.25–0.12]
Place of residence (Rural)	-0.25***[-0.36–0.15]	- -	-0.25***[-0.36–0.15]
Attending private pre-school (endogenous variable)			
Mother's education	0.04**[0.01–0.07]	- -	0.04**[0.01–0.07]
Father's education	0.03[0.00–0.06]	- -	0.03[0.00–0.06]
Wealth index	0.06***[0.04–0.08]	- -	0.06***[0.04–0.08]
Caste (SC/ST)	0.03[-0.04–0.10]	- -	0.03[-0.04–0.10]
Religion (Non-Hindu)	-0.13***[-0.19–0.06]	- -	-0.13***[-0.19–0.06]
Place of residence (Rural)	0.04[-0.06–0.15]	- -	0.04[-0.06–0.15]
ECD score (dependent variable)			
HE score	0.46***[0.38–0.55]	- -	0.46***[0.38–0.55]
Mother's time for stimulating activities	0.41***[0.22–0.61]	- -	0.41***[0.22–0.61]
Home tuition (Yes)	0.43***[0.17–0.70]	- -	0.43***[0.17–0.70]
Attending private pre-school (Yes)	0.76***[0.43–1.09]	- -	0.76***[0.43–1.09]
Child's age	0.06***[0.05–0.07]	0.01***[0.01–0.02]	0.07***[0.06–0.09]
Child's sex (Female)	0.05[-0.13–0.23]	0.10*[0.02–0.18]	0.15[-0.05–0.34]
Birth order	0.00[-0.09–0.10]	-0.05*[-0.09–0.01]	-0.05[-0.15–0.05]
Mother's education	0.13*[0.02–0.25]	0.30***[0.22–0.38]	0.43***[0.31–0.56]
Father's education	0.19***[0.08–0.30]	0.08**[0.02–0.13]	0.26***[0.15–0.38]
Mother's happiness	-0.07[-0.17–0.02]	-0.02[-0.06–0.01]	-0.10[-0.20–0.00]
Wealth index	0.02[-0.05–0.09]	0.13***[0.09–0.17]	0.15***[0.08–0.22]
Caste (SC/ST)	-0.29*[-0.54–0.04]	0.03[-0.1–0.17]	-0.25[-0.53–0.03]
Religion (Non-Hindu)	0.23*[0.00–0.47]	-0.48***[-0.62–0.33]	-0.25[-0.50–0.01]

(*Continued*)

Table 2. (Continued)

Characteristics	Direct effects	Indirect effects	Total effects
	**Coef. [95% CI]**	**Coef. [95% CI]**	**Coef. [95% CI]**
Place of residence (Rural)	-0.47*[-0.84–0.09]	-0.40***[-0.62–0.19]	-0.87***[-1.28–0.46]
Attending AWCs/ICDS	-0.30*[-0.56–0.04]	- -	-0.30*[-0.56–0.04]
Father's time for stimulating activities	0.15[-0.16–0.46]	- -	0.15[-0.16–0.46]
Stunting (Yes)	-0.07[-0.27–0.12]	- -	-0.07[-0.27–0.12]
Mother's occupation (Bidi/agri. worker)	0.09[-0.13–0.31]	-0.11*[-0.21–0.01]	-0.02[-0.26–0.22]
Domestic violence (Yes)	-0.27[-0.55–0.02]	- -	-0.27[-0.55–0.02]

Note:

*** significant level at *p* value = <0.001,

** significant level at *p* value = <0.01, and

* significant level at *p* value = <0.05; CI = Confidence Intervals; Coef. = Coefficients.

Sources: Primary field survey, Malda, 2018.

https://doi.org/10.1371/journal.pone.0268985.t002

the indirect path, largely through the home environment. A surprising finding is observed in the case of the religious groups in the path model. The non-Hindu religious group is not significantly associated with ECD shown by total effects, but positively correlated with ECD by direct path (Coef. 0.23, $p = <0.050$), and significantly negatively correlated with the same trough indirect path (Coef. -0.48, $p = <0.001$). All the endogenous variables are negatively associated with the non-Hindu religious group. Among them, mother's time for care is negatively influenced mostly.

On the other hand, attending AWCs is negatively (Coef. -0.30, $p = <0.050$) associated with ECD, shown by total effects.

Among the exogenous factors, place of residence has the strongest effect on ECD. The total effect of rural areas as the place of residence is the highest one (Coef. -0.87, $p = <0.001$) in the path model, and this is highly negatively correlated to ECD. The larger part of the effect of the place of residence reflects through the direct path (Coef. -0.47, $p = <0.050$), and the remaining part of the effect (Coef. -0.40, $p = <0.001$) reflects through the indirect way via home environment, mother's time for care and home tuition. Rural areas affect the mother's time for care mostly among other endogenous variables.

Among the endogenous factors, the type of pre-schools has the highest effect on ECD. Attending private pre-school is highly positively (Coef. 0.76, $p = <0.001$) correlated with ECD. Attending private pre-school is largely influenced by religion, followed by the mother's education and wealth index. As compared to the Hindus, Muslims/others are negatively associated with attending private pre-school, whereas the mother's education and wealth index are positively associated with the same. Having home tuition is another important factor of ECD, positively (Coef. 0.43, $p = <0.001$) correlated with ECD. Having home tuition is largely determined by the place of residence and religion. As compared to Hindus and urban residence, Muslims and rural residence, respectively, are negatively associated with home tuition. Another important factor of ECD status is the mother's time for stimulating activities, positively correlated (Coef. 0.41, $p = <0.001$) with ECD. Mother's time devoted for stimulating activities is mostly controlled by the place of residence, religion, caste groups and mother's education. Rural residence, Muslims/others and non-SC/ST are negatively associated with the mother's time for stimulating activities, whereas the mother's education is positively associated with the same.

4. Discussion

Results of this study showed that only about 25% of children are developmentally on track of literacy-numeracy domain of ECD. In other words, it can be said that three children in every four children are not at the expected level of ECD in the literacy-numeracy domain as per UNICEF's measure [46]. In this regard, a study based in 35 developing countries using the recent round of MICS data has revealed that 29.9% of children are developmentally on track in the same domain [115]. Therefore, it can be said that the condition in this domain is worse for Malda district in comparison to the other developing countries. Although, in the other domains, the situation is better. For example, about 83% of children are developmentally on track in the learning domain, almost 95% and 99% of children are developmentally on track in social-emotional and physical fitness domains, respectively.

By and large, it has been seen that with the increase in mothers' and fathers' level of education, the status of ECD also increases. It is also found that the children belonging to joint families and Hindu households are more advanced in the process of ECD status than the children belong to single families and Muslim/other households, respectively. It has also been found that the status of ECD is higher for the children of the non-ST/SC category than their counterparts, ST/SC category. Further, it has been seen that the children living in rural areas perform very poorly in the process of ECD, where the mean score of ECD is also almost half than that of the children living in urban areas. It has also been observed that the children who are attending private pre-schools, having home tuitions are more advanced in the process of ECD than their respective counterparts.

The results of the path analysis revealed that there are certain mediating factors or proximal factors, i.e., home environment, mother's time for care, having home tuition and attending private pre-school, that are influenced by certain distal or exogenous factors and subsequently affecting the outcome measure or the process of ECD. It has been found that the total effects (direct effect + indirect effect) of the place of residence was the highest one in the path model. The second most influential factor was attending private pre-school followed by having home tuition, mother's education level, and mother's time for various stimulating activities, father's education and household income (wealth index) as shown by total effects. Place of residence affects the process of ECD mostly through mothers' time for stimulating activities, then through home environment and home tuition. In general, mothers/caregivers from urban areas are more educated and more aware of their children's health and academic achievement. Thus, they foster a more favourable home environment and give more time to their children in various stimulating activates. They are also more likely to provide home tuition for their children, which altogether help in the overall development of their children than the children of mothers from rural areas. It has been observed that household income (wealth index) influences the process of ECD through home environment mostly, then through attending private pre-school and having home tuition. This finding is partially consistent with the latest study based in the Ghanaian context [92]. Obviously, wealthier households are more capable of providing books and playing materials to their children. Again, parents or other members of the affluent households are more likely to engage in various stimulating activities with the children, which may be because of their higher education status and higher expectations from the children. Further, they are more capable of affording the cost of home tuitions and private preschooling, which altogether adds substantially to the process of ECD as compared to the children belong to households of lower economic status. Parental education was also an important factor of ECD. Most of the fathers' education effect on ECD was direct, whereas most of the mothers' education effect on ECD was indirect. Mothers'/caregivers' education influences the process of ECD via home environment largely, then, through mothers' time for care, private

pre-schooling and having home tuition. The recent Ghanaian study also found that parental education affects children's outcomes positively in various ways [92]. In Indian society, mothers are the primary caregivers for their children, and they usually spend more time daily with children than fathers. Therefore, if the mothers who are higher educated create a favourable home environment and engage more in various stimulating activities for a longer duration with their children, that enhances the ECD status of their children as compared to the children of mothers with lower educational status. A conspicuous finding in the path model was that the non-Hindus as the Muslim or other minor religious group, positively correlated with ECD directly, while this was negatively correlated with the same indirectly, and the total effect was also negative but statistically insignificant. This may be due to the fact that children of deprived communities, i.e., ST and SC, are also placed under the Hindu religion, and their performance is very poor in the domains of ECD. This is the possible explanation of why the children of non-Hindu families are doing better in the process of ECD in a direct way. However, non-Hindu religion affects the process of ECD negatively through the indirect path, where most of the negative effect is accounted by mothers' time for care followed by the home environment, home tuition and attending private pre-school. Thus, from this finding, it can be stated that children of Muslim families can also perform better in the process of ECD if they get the same home environment, care, access to private pre-schools as that availed by the children of Hindu families. Another striking finding in the path model was attending AWCs of ICDS schemes, negatively associated with ECD. While one of the objectives of ICDS is *"to lay the foundation for proper psychological, physical and social development of the child"*, which had been launched in 1975 in India [116]. The reason for this negative association can be explained by the fact that the majority of children who are attending the AWCs belong to socio-economically disadvantaged households. Also, the quality of services, i.e., poor services and poor infrastructures, un-skilled Anganwadi workers (AWWs), poor administration and lack of proper monitoring or evaluation of ICDS programmes might be possible reasons for this negative association.

There are some limitations in this study, which need to be mentioned here. Since the study's dataset was based on a cross-sectional survey, the results of this study are non-causal. Also, the indicators of home environment and ECD were assessed based on mothers or caregivers' observations that can be biased or not accurate. Notwithstanding, this study has revealed that the home environment created by the parents has a significant contribution to ECD, which is already established in the literature. However, the present research contributes to the existing knowledge of the ECD that mothers' time for various stimulating activities with their children, fetching home tuition for children and children attending private pre-school, are the decisive influential factors of ECD in the Indian context.

5. Conclusion

An attempt has been made in this study to assess ECD status in Malda district that is one of the backward districts of West Bengal, India. This study revealed that ECD status is very poor, particularly in literacy and numeracy domains of development. Even the situation in these domains is worse in Malda district as compared to the level of development in the same domains in developing countries. Thus, this is a matter of concern. Urgent attention from policymakers or other stakeholders is needed to promote ECD for the betterment of children and society of Malda district. Further, a conceptual framework has been built to examine a complex relationship and to test the hypothesis that the quality of home environment or parenting practice influences ECD largely. Also, it has been assumed that children's home environment will be influenced by parent's socio-economic characteristics and child characteristics. Accordingly, this study has examined the relationship between ECD and home environment, children

attending AWCs/ICDS and not attending or attending other pre-schools, having home tuition or not, and the time given by the parents for various stimulating activities with other exogenous factors. The results of this research have established that the place of residence, whether rural or urban areas, and attending private pre-school are influencing ECD largely rather than the quality of home environment. Although, it has also been realised that the quality of home environment that is controlled by certain parent's socio-economic characteristics, a crucial factor for ECD in this study. Also, it has been found that having home tuition is an important factor for ECD, whereas attending public pre-schools or AWCs is negatively associated with ECD. From these findings, it is recommended that the existing ICDS programmes should be monitored and evaluated regularly as most of the children are accessing AWCs; on the other hand, the affordability and accessibility to private pre-schools and home tuition are not possible for the majority of the parents because of their poor socio-economic condition in this study setting. Thus, the quality of services in the ICDS programmes needs to be improved. Parental time for various stimulating activities with their children, especially which allocated by mothers, is another vital factor for ECD. Therefore, both parents should be encouraged to invest more time with their children, which will enhance the ECD status. In addition to these, special attention needs to be given to the children belonging to Muslim families and children belonging to socially deprived communities (e.g., SC and ST) since they are less advanced in the process of ECD as compared to the children belonging to the Hindu families and non-SC/ST communities, respectively.

Supporting information

S1 Data.
(DTA)

Acknowledgments

I am very grateful to Prof. Anuradha Banerjee, Centre for the Study of Regional Development, Jawaharlal Nehru University, New Delhi, for his overall supervision in preparing the manuscript. I am also thankful to Prof. KS James, International Institute for Population Sciences, Mumbai and Ret. Prof. PM Kulkarni, Jawaharlal Nehru University, New Delhi for helping in the sampling framework for this research.

Author Contributions

Conceptualization: Rayhan Sk.

Data curation: Rayhan Sk.

Formal analysis: Rayhan Sk.

Funding acquisition: Rayhan Sk.

Investigation: Rayhan Sk.

Methodology: Rayhan Sk.

Project administration: Rayhan Sk.

Resources: Rayhan Sk.

Software: Rayhan Sk.

Writing – original draft: Rayhan Sk.

Writing – review & editing: Rayhan Sk.

References

1. Shonkoff JP, Richmond JB. Investment in Early Childhood Development Lays the Foundation for a Prosperous and Sustainable Society. Encycl Early Child Dev [online] [Internet]. 2009;1–4. http://www.child-encyclopedia.com/sites/default/files/textes-experts/en/669/investment-in-early-childhood-development-lays-the-foundation-for-a-prosperous-and-sustainable-society.pdf
2. Lo S, Das P, Horton R. Early childhood development: the foundation of sustainable. 2016;(2011):9–11. https://ac.els-cdn.com/S0140673616316592/1-s2.0-S0140673616316592-main.pdf?_tid=91a45721-ff0a-4f0c-9eca-d7de4e50b719&acdnat=1552399716_ba0c323cc659c9cc88a39f5a93636739
3. Lu C, Black MM, Richter LM. Articles Risk of poor development in young children in low-income and middle-income countries: an estimation and analysis at the global, regional, and country level. Lancet Glob Heal [Internet]. 2016; 4(12):e916–22. Available from: www.thelancet.com/lancetgh
4. Knudsen EI, Heckman JJ, Cameron JL, Shonkoff JP. Economic, neurobiological, and behavioral perspectives on building America's future workforce. Proc Natl Acad Sci [Internet]. 2006 Jul 5; 103 (27):10155–62. Available from: http://www.pnas.org/cgi/doi/10.1073/pnas.0600888103 PMID: 16801553
5. Irwin L, Siddiqi A, Hertzman C. Early Child Development: A Powefull Equalizer. Final Report [Internet]. Geneav; 2007. https://www.who.int/social_determinants/resources/ecd_kn_report_07_2007.pdf
6. Black MM, Walker SP, Fernald LCH, Andersen CT, Digirolamo AM, Lu C, et al. Advancing Early Childhood Development: from Science to Scale 1 Early childhood development coming of age: science through the life course. 2017.
7. McCoy DC, Peet ED, Ezzati M, Danaei G, Black MM, Sudfeld CR, et al. Early Childhood Developmental Status in Low- and Middle-Income Countries: National, Regional, and Global Prevalence Estimates Using Predictive Modeling. PLoS Med. 2016; 13(6):1–18.
8. United Nation. Sustainable Development Goals. 2015.
9. Every Woman Every Child. The Global Strategy for Women's, Children's and Adolescents' Health (2016–2030) [Internet]. Every Woman Every Childl. Italy; 2015. https://www.who.int/life-course/partners/global-strategy/globalstrategyreport2016-2030-lowres.pdf
10. Grantham-McGregor S, Cheung YB, Cueto S, Glewwe P, Richter L, Strupp B. Developmental potential in the first 5 years for children in developing countries. Lancet. 2007; 369(9555):60–70. https://doi.org/10.1016/S0140-6736(07)60032-4 PMID: 17208643
11. Danaei G, Andrews KG, Sudfeld CR, Fink G, McCoy DC, Peet E, et al. Risk Factors for Childhood Stunting in 137 Developing Countries: A Comparative Risk Assessment Analysis at Global, Regional, and Country Levels. Tumwine JK, editor. PLOS Med [Internet]. 2016 Nov 1; 13(11):e1002164. Available from: https://dx.plos.org/10.1371/journal.pmed.1002164 PMID: 27802277
12. Walker SP, Wachs TD, Grantham-McGregor S, Black MM, Nelson CA, Huffman SL, et al. Inequality in early childhood: risk and protective factors for early child development. Lancet [Internet]. 2011 Oct; 378(9799):1325–38. Available from: https://linkinghub.elsevier.com/retrieve/pii/S0140673611605552 PMID: 21944375
13. Grantham-McGregor SM, Fernald LCH, Kagawa RMC, Walker S. Effects of integrated child development and nutrition interventions on child development and nutritional status. Ann N Y Acad Sci [Internet]. 2014 Jan; 1308(1):11–32. Available from: http://doi.wiley.com/10.1111/nyas.12284 PMID: 24673166
14. Engle PL, Fernald LC, Alderman H, Behrman J, O'Gara C, Yousafzai A, et al. Strategies for reducing inequalities and improving developmental outcomes for young children in low-income and middle-income countries. Lancet [Internet]. 2011 Oct; 378(9799):1339–53. Available from: http://dx.doi.org/10.1016/S0140-6736(11)60889-1 PMID: 21944378
15. Engle PL, Black MM, Behrman JR, Cabral de Mello M, Gertler PJ, Kapiriri L, et al. Strategies to avoid the loss of developmental potential in more than 200 million children in the developing world. Lancet [Internet]. 2007 Jan 1; 369(9557):229–42. Available from: https://www.thelancet.com/action/showPdf?pii=S0140-6736%2807%2960112-3 PMID: 17240290
16. Registrar General of India. Census of India: Age Structure And Marital Status [Internet]. 2011. http://censusindia.gov.in/Census_And_You/age_structure_and_marital_status.aspx
17. Registrar General of India. Primary Census Abstrat. New Delhi; 2011.

18. United Nations, Department of Economic and Social Affairs PD. World Population Prospects The 2017 Revision [Internet]. New York; 2017. https://esa.un.org/unpd/wpp/publications/files/wpp2017_keyfindings.pdf

19. Bronfenbrenner U. The Ecology of Human Develpment. Experiments by nature and design [Internet]. London, England: Harvard University Press; 1979. 330 p. https://khoerulanwarbk.files.wordpress.com/2015/08/urie_bronfenbrenner_the_ecology_of_human_developbokos-z1.pdf PMID: 83896

20. Bornstein MH, Leventhal T. Children in Bioecological Landscapes of Development. Handb Child Psychol Dev Sci. 2015; 4:1–5.

21. Bronfenbrenner U. Bronfenbrenner U. (1994). Ecological models of human devleopment. In International Encyclopedia of Education. Vol 3. 2nd Ed. Pxford: Elsevier. Int Encycl Educ. 1994;3.

22. Bronfenbrenner U, Morris PA. The Bioecological Model of Human Development. In: Handbook of Child Psychology [Internet]. Hoboken, NJ, USA: John Wiley & Sons, Inc.; 2007. p. 085201. https://www.taylorfrancis.com/books/9781317294313/chapters/10.4324/9781315646206-2

23. Shonkoff JP. Building a New Biodevelopmental Framework to Guide the Future of Early Childhood Policy. 2010; 81(1):357–67.

24. Duckworth K, Sabates R. Effects of mothers ' education on parenting: an investigation across three generations. London Rev Educ. 2005; 3(3):239–64.

25. Orth U. The family environment in early childhood has a long-term effect on self-esteem: A longitudinal study from birth to age 27 years. J Pers Soc Psychol [Internet]. 2018 Apr; 114(4):637–55. Available from: http://dx.doi.org/10.1037/pspp0000143 PMID: 28182449

26. dos Santos LM, dos Santos DN, Bastos ACS, Assis AMO, Prado MS, Barreto ML. Determinants of early cognitive development: hierarchical analysis of a longitudinal study. Cad Saude Publica. 2008; 24(2):427–37. https://doi.org/10.1590/s0102-311x2008000200022 PMID: 18278290

27. Sun J, Liu Y, Chen EE, Rao N, Liu H. Early Childhood Research Quarterly Factors related to parents ' engagement in cognitive and socio-emotional caregiving in developing countries: Results from Multiple Indicator Cluster Survey 3. Early Child Res Q [Internet]. 2016; 36:21–31. Available from: http://dx.doi.org/10.1016/j.ecresq.2015.12.003

28. Walker SP, Wachs TD, Gardner JM, Lozoff B, Wasserman GA, Pollitt E, et al. Child development: risk factors for adverse outcomes in developing countries. Lancet [Internet]. 2007 Jan 13; 369 (9556):145–57. Available from: https://linkinghub.elsevier.com/retrieve/pii/0315086079901307 PMID: 17223478

29. Luster T, Okagaki L. Parenting: An Ecological Perspective [Internet]. Mahwah, NJ, US: Lawrence Erlbaum Associates; 2005. https://books.google.co.in/books?id=wLNTNItwJZUC

30. Bradley RH, Corwyn RF, Mcadoo HP, Coll CG. The Home Environments of Children in the United States Part I: Variations by Age, Ethnicity, and Poverty Status. 2001; 72(6):1844–67.

31. Belsky J. The Determinants of Parenting: A Process Model. Child Dev [Internet]. 1984 Feb; 55(1):83. Available from: https://www.jstor.org/stable/1129836?origin=crossref PMID: 6705636

32. Belsky J, Robins E, Gamble W. The Determinants of Parental Competence. In: Beyond The Dyad [Internet]. Boston, MA: Springer US; 1984. p. 251–79. http://link.springer.com/10.1007/978-1-4757-9415-1_11

33. Baharuddin Rozumah and Luster T. Factors Related to the Quality of the Home Environment and Children's Achivement. J Fam Issues. 1998; 19(4):375–403.

34. Elardo R, Bradley R, Caldwell BM. The Relation of Infants ' Home Environments to Mental Test Performance from Six to Thirty-Six Months: A Longitudinal Analysis Author (s): Richard Elardo, Robert Bradley and Bettye M. Caldwell Published by: Wiley on behalf of the Society for Research. Child Dev [Internet]. 1975; 46(1):71–6. Available from: https://www.jstor.org/stable/1128835https://www.jstor.org/stable/1128835

35. Yousafzai AK, Obradović J, Rasheed MA, Rizvi A, Portilla XA, Tirado-Strayer N, et al. Effects of responsive stimulation and nutrition interventions on children's development and growth at age 4 years in a disadvantaged population in Pakistan: a longitudinal follow-up of a cluster-randomised factorial effectiveness trial. Lancet Glob Heal [Internet]. 2016 Aug; 4(8):e548–58. Available from: https://linkinghub.elsevier.com/retrieve/pii/S2214109X16301000

36. Totsika V, Sylva K. The Home Observation for Measurement of the Environment Revisited. Child Adolesc Ment Health [Internet]. 2004 Feb; 9(1):25–35. Available from: http://link.springer.com/10.1007/978-3-319-13942-5_21 PMID: 32797621

37. Harden BJ, Whittaker JV. The early home environment and developmental outcomes for young children in the child welfare system. Child Youth Serv Rev [Internet]. 2011 Aug; 33(8):1392–403. Available from: http://dx.doi.org/10.1016/j.childyouth.2011.04.009

38. Biedinger N. The Influence of Education and Home Environment on the Cognitive Outcomes of Preschool Children in Germany. Child Dev Res [Internet]. 2011; 2011:1–10. Available from: http://www.hindawi.com/journals/cdr/2011/916303/

39. Kaushal S, Singh CK, Rana MK. Influence of Home Environment on Psychological Abilities of Children. Stud Home Community Sci [Internet]. 2013 Dec; 7(3):157–60. Available from: https://www.tandfonline.com/doi/full/10.1080/09737189.2013.11885407

40. Nguyen PH, DiGirolamo AM, Gonzalez-Casanova I, Young M, Kim N, Nguyen S, et al. Influences of early child nutritional status and home learning environment on child development in Vietnam. Matern Child Nutr [Internet]. 2018 Jan; 14(1):e12468. Available from: http://doi.wiley.com/10.1111/mcn.12468 PMID: 28585371

41. McLoyd VC. The Impact of Economic Hardship on Black Families and Children: Psychological Distress, Parenting, and Socioemotional Development. Child Dev [Internet]. 1990 Apr; 61(2):311–46. Available from: http://doi.wiley.com/10.1111/j.1467-8624.1990.tb02781.x PMID: 2188806

42. Hannan K, Luster T. Influence of parent, child, and contextual factors on the quality of the home environment. Infant Ment Health J [Internet]. 1991; 12(1):17–30. Available from: http://doi.wiley.com/10.1002/1097-0355%28199121%2912%3A1%3C17%3A%3AAID-IMHJ2280120103%3E3.0.CO%3B2-3

43. Menaghan EG, Parcel TL. Determining Children's Home Environments: The Impact of Maternal Characteristics and Current Occupational and Family Conditions. J Marriage Fam [Internet]. 1991 May; 53(2):417. Available from: https://www.jstor.org/stable/352909?origin=crossref

44. Bradley RH, Caldwell BM, Rock SL, Hamrick HM, Harris P. Home Observation for Measurement of the Environment: Development of a Home Inventory for use with families having children 6 to 10 years old. Contemp Educ Psychol [Internet]. 1988 Jan; 13(1):58–71. Available from: https://linkinghub.elsevier.com/retrieve/pii/0361476X88900069

45. Bronfenbrenner U, Alvarez WF, Henderson CR. Working and Watching: Maternal Employment Status and Parents' Perceptions of Their Three-Year-Old Children. Child Dev [Internet]. 1984 Aug; 55(4):1362. Available from: https://www.jstor.org/stable/1130006?origin=crossref

46. UNICEF. The formative years: UNICEF ' s work on measuring early childhood development [Internet]. New York; 2014. http://data.unicef.org/wp-content/uploads/2015/12/Measuring-ECD-Brochure-HR-10_8_116.pdf

47. Frongillo EA, Kulkarni S, Basnet S, de Castro F. Family Care Behaviors and Early Childhood Development in Low- and Middle-Income Countries. J Child Fam Stud [Internet]. 2017 Nov 13; 26(11):3036–44. Available from: http://dx.doi.org/10.1007/s10826-017-0816-3

48. Jeong J, McCoy DC, Fink G. Pathways between paternal and maternal education, caregivers' support for learning, and early child development in 44 low- and middle-income countries. Early Child Res Q [Internet]. 2017; 41(July):136–48. Available from: http://dx.doi.org/10.1016/j.ecresq.2017.07.001

49. Jeong J, Mccoy C, Yousafzai AK, Salhi C, Fink G. Paternal Stimulation and Early Child Development in Low- and Middle-Income Countries. 2016; 138(4).

50. Sk R, Banerjee A, Mishra R, Barua S. Quality of care and early childhood developmental status in Nepal: a multilevel analysis. Early Child Dev Care [Internet]. 2019 Jan 23;1–14. Available from: https://www.tandfonline.com/doi/full/10.1080/03004430.2019.1570503

51. Wertsch JV, McNamee GD, McLane JB, Budwig NA. The Adult-Child Dyad as a Problem-Solving System. Child Dev [Internet]. 1980 Dec; 51(4):1215. Available from: https://www.jstor.org/stable/1129563?origin=crossref

52. Rowe ML, Pan BA, Ayoub C. Predictors of Variation in Maternal Talk to Children: A Longitudinal Study of Low-Income Families. Parenting [Internet]. 2005 Aug; 5(3):259–83. Available from: http://www.tandfonline.com/doi/abs/10.1207/s15327922par0503_3

53. Ivrendi A, Isikoglu N. A Turkish View on Fathers' Involvement in Children's Play. Early Child Educ J [Internet]. 2010 Apr 6; 37(6):519–26. Available from: http://link.springer.com/10.1007/s10643-010-0376-2

54. Leaper C, Anderson KJ, Sanders P. Moderators of gender effects on parents' talk to their children: A meta-analysis. Dev Psychol [Internet]. 1998; 34(1):3–27. Available from: http://doi.apa.org/getdoi.cfm?doi=10.1037/0012-1649.34.1.3 PMID: 9471001

55. Eisenman R. Birth order, development and personality. Acta Paedopsychiatr Int J Child Adolesc Psychiatr. 1992; 55(1):25–7. PMID: 1310365

56. Sputa CL, Paulson SE. Birth Order and Family Size: Influences on Adolescents' Achievement and Related Parenting Behaviors. Psychol Rep [Internet]. 1995 Feb; 76(1):43–51. Available from: http://journals.sagepub.com/doi/10.2466/pr0.1995.76.1.43 PMID: 7770592

57. Iacovou M. Family Size, Birth Order, and Educational Attainment. Marriage Fam Rev [Internet]. 2008 Jan 8; 42(3):35–57. Available from: http://www.tandfonline.com/doi/abs/10.1300/J002v42n03_03

58. WHO. PRETERM BIRTH. 2018.

59. Moreira RS, Magalhães LC, Alves CRL. Effect of preterm birth on motor development, behavior, and school performance of school-age children: a systematic review. J Pediatr (Rio J) [Internet]. 2014 Mar; 90(2):119–34. Available from: http://dx.doi.org/10.1016/j.jped.2013.05.010

60. Beauregard JL, Drews-Botsch C, Sales JM, Flanders WD, Kramer MR. Preterm Birth, Poverty, and Cognitive Development. Pediatrics [Internet]. 2018 Jan; 141(1):e20170509. Available from: http://pediatrics.aappublications.org/lookup/doi/10.1542/peds.2017-0509 PMID: 29242268

61. Ip P, Ho FKW, Rao N, Sun J, Young ME, Chow CB, et al. Impact of nutritional supplements on cognitive development of children in developing countries: A meta-analysis. Sci Rep [Internet]. 2017 Dec 6; 7(1):10611. Available from: http://dx.doi.org/10.1038/s41598-017-11023-4 PMID: 28878390

62. Huiracocha-Tutiven L, Orellana-Paucar A, Abril-Ulloa V, Huiracocha-Tutiven M, Palacios-Santana G, Blume S. Child Development and Nutritional Status in Ecuador. Glob Pediatr Heal [Internet]. 2019 Jan 23; 6:2333794X1882194. Available from: http://journals.sagepub.com/doi/10.1177/2333794X18821946 PMID: 30719492

63. Jimoh AO, Anyiam JO, Yakubu AM. Relationship between child development and nutritional status of under-five Nigerian children. South African J Clin Nutr [Internet]. 2018 Jul 3; 31(3):50–4. Available from: http://doi.org/10.1080/16070658.2017.1387434

64. Warsito O, Khomsan A, Hernawati N, Anwar F. Relationship between nutritional status, psychosocial stimulation, and cognitive development in preschool children in Indonesia. Nutr Res Pract [Internet]. 2012; 6(5):451. Available from: http://www.hindawi.com/journals/jnm/2015/452148/ PMID: 23198025

65. Maggi S, Irwin LJ, Siddiqi A, Hertzman C. The social determinants of early child development: An overview. J Paediatr Child Health [Internet]. 2010 Nov; 46(11):627–35. Available from: http://doi.wiley.com/10.1111/j.1440-1754.2010.01817.x PMID: 20796183

66. Pelto GH, Dickin K, Engle PL. A Critical Link- Interventions for Physical Growth and Psychological Development. Nutr Anthropol [Internet]. 1999; 22(2):21–7. Available from: http://doi.wiley.com/10.1525/nua.1999.22.2.21

67. Liaw F, Brooks-gunn J, Liaw F, Brooks-gunn J. Cumulative familial risks and low-birthweight children ' s cognitive and behavioral development Cumulative Familial Risks and Low-Birthweight Children ' s Cognitive and Behavioral Development. J Clin Child Psychol. 1994; 23(4):360–272.

68. Maggi S, Irwin LG, Siddiqi A, Poureslami I, Hertzman E, Hertzman C. 'Knowledge Network for Early Childhood Development: Analytic and strategic review paper. International perspectives on early childhood development [Internet]. Initiatives. 2005. https://www.who.int/social_determinants/resources/ecd.pdf?ua=1

69. Dickson M, Gregg P, Robinson H. Early, Late or Never? When Does Parental Education Impact Child Outcomes? Econ J [Internet]. 2016 Oct 1; 126(596):F184–231. Available from: https://academic.oup.com/ej/article/126/596/F184-F231/5077841

70. Duncan GJ, Magnuson K, Votruba-Drzal E. Boosting Family Income to Promote Child Development. Futur Child [Internet]. 2014; 24(1):99–120. Available from: http://muse.jhu.edu/content/crossref/journals/future_of_children/v024/24.1.duncan.html PMID: 25518705

71. Duncan GJ, Haveman R, Wolfe B. Succeeding Generations: On the Effects of Investments in Children. J Policy Anal Manag [Internet]. 1995; 14(4):639. Available from: https://www.jstor.org/stable/3324918?origin=crossref

72. Ertem IO, Atay G, Dogan DG, Bayhan A, Bingoler BE, Gok CG, et al. Mothers' knowledge of young child development in a developing country. Child Care Health Dev [Internet]. 2007 Nov; 33(6):728–37. Available from: http://doi.wiley.com/10.1111/j.1365-2214.2007.00751.x PMID: 17944782

73. Schady N. Parents' Education, Mothers' Vocabulary, and Cognitive Development in Early Childhood: Longitudinal Evidence From Ecuador. Am J Public Health [Internet]. 2011 Dec; 101(12):2299–307. Available from: http://ajph.aphapublications.org/doi/10.2105/AJPH.2011.300253 PMID: 22021308

74. Prickett KC, Augustine JM. Maternal Education and Investments in Children's Health. J Marriage Fam [Internet]. 2016 Feb; 78(1):7–25. Available from: http://doi.wiley.com/10.1111/jomf.12253 PMID: 26778853

75. Davis-Kean PE. The Influence of Parent Education and Family Income on Child Achievement: The Indirect Role of Parental Expectations and the Home Environment. J Fam Psychol [Internet]. 2005; 19(2):294–304. Available from: http://doi.apa.org/getdoi.cfm?doi=10.1037/0893-3200.19.2.294 PMID: 15982107

76. Hamadani AJD. Cognitive De fi cit and Poverty in the First 5 Years of Childhood in Bangladesh. 2014; 134(4).

77. Rubio-Codina M, Attanasio O, Grantham-McGregor S. Mediating pathways in the socio-economic gradient of child development. Int J Behav Dev [Internet]. 2016 Nov 9; 40(6):483–91. Available from: http://journals.sagepub.com/doi/10.1177/0165025415626515 PMID: 27885311

78. Augustine JM. Mothers ' Employment, Education, and Parenting. Work Occup [Internet]. 2014 May 24; 41(2):237–70. Available from: http://journals.sagepub.com/doi/10.1177/0730888413501342

79. Reynolds SA, Fernald LCH, Behrman JR. Mothers' labor market choices and child development outcomes in Chile. SSM—Popul Heal [Internet]. 2017 Dec; 3(December 2016):756–66. Available from: https://doi.org/10.1016/j.ssmph.2017.08.010 PMID: 29349262

80. Kalmijn M. Mother's Occupational Status and Children's Schooling. Am Sociol Rev [Internet]. 1994 Apr; 59(2):257–75. Available from: http://www.jstor.org/stable/2096230?origin=crossref

81. Youn MJ, Leon J, Lee KJ. The influence of maternal employment on children's learning growth and the role of parental involvement. Early Child Dev Care [Internet]. 2012 Sep; 182(9):1227–46. Available from: http://www.tandfonline.com/doi/abs/10.1080/03004430.2011.604944

82. Raver CC. Does Work Pay Psychologically as Well as Economically? The Role of Employment in Predicting Depressive Symptoms and Parenting Among Low-Income Families. Child Dev [Internet]. 2003 Nov; 74(6):1720–36. Available from: http://doi.wiley.com/10.1046/j.1467-8624.2003.00634.x PMID: 14669892

83. Menaghan EG, Parcel TL. Social Sources of Change in Children's Home Environments: The Effects of Parental Occupational Experiences and Family Conditions. J Marriage Fam [Internet]. 1995 Feb; 57 (1):6984. Available from: https://www.jstor.org/stable/353817?origin=crossref

84. Osofsky JD. The effect of exposure to violence on young children. In: American Psychologist [Internet]. 1995. p. 782–8. http://doi.apa.org/getdoi.cfm?doi=10.1037/0003-066X.50.9.782 PMID: 7574189

85. Wathen CN, Macmillan HL. Children's exposure to intimate partner violence: Impacts and interventions. Paediatr Child Health [Internet]. 2013 Oct; 18(8):419–22. Available from: http://www.ncbi.nlm.nih.gov/pubmed/24426794 PMID: 24426794

86. Margolin G, Gordis EB. The Effects of Family and Community Violence on Children. Annu Rev Psychol [Internet]. 2000 Feb; 51(1):445–79. Available from: https://linkinghub.elsevier.com/retrieve/pii/S0016508514601451

87. Kolbo JR, Blakely EH, Engleman D. Children Who Witness Domestic Violence: A Review of Empirical Literature. J Interpers Violence [Internet]. 1996 Jun 2; 11(2):281–93. Available from: http://journals.sagepub.com/doi/10.1177/016344300022005001

88. Fantuzzo JW, Lindquist CU. The effects of observing conjugal violence on children: A review and analysis of research methodology. J Fam Violence [Internet]. 1989 Mar; 4(1):77–94. Available from: http://link.springer.com/10.1007/BF00985658

89. Kitzmann KM. Domestic Violence and Its Impact on the Social and Emotional Development of Young Children. Development. 2007;(May 2005):1–7.

90. Ajilchi B, Kargar FIR, Ghoreishi MK. Relationship between the Parenting Styles of Overstressed Mothers with their Children's Self-esteem. Procedia—Soc Behav Sci [Internet]. 2013 Jul; 82:496–501. Available from: http://dx.doi.org/10.1016/j.sbspro.2013.06.299

91. Bahrami A. The Effect of Happiness on Various Aspects of Parenting and Positive Parenting. Psychol Behav Sci Int J [Internet]. 2017 May 23; 4(1):1–6. Available from: https://juniperpublishers.com/pbsij/PBSIJ.MS.ID.555627.php

92. Wolf S, McCoy DC. Household Socioeconomic Status and Parental Investments: Direct and Indirect Relations With School Readiness in Ghana. Child Dev. 2019; 90(1):260–78. https://doi.org/10.1111/cdev.12899 PMID: 28708296

93. Birch HG. Nutrition, growth, and mental development—eightieth annual meeting of American pediatric society introductory remarks. Am J Dis Child. 1970; 120(5):395–7.

94. Nievar MA, Luster T. Developmental Processes in African American Families: An Application of McLoyd's Theoretical Model. J Marriage Fam [Internet]. 2006 May; 68(2):320–31. Available from: http://doi.wiley.com/10.1111/j.1741-3737.2006.00255.x

95. Duncan GJ, Brooks-Gunn J, Klebanov PK. Economic Deprivation and Early Childhood Development. Child Dev [Internet]. 1994 Apr; 65(2):296. Available from: https://www.jstor.org/stable/1131385?origin=crossref PMID: 7516849

96. Armistead L, Forehand R, Brody G, Maguen S. Parenting and child psychosocial adjustment in single-parent African American families: Is community context important? Behav Ther [Internet]. 2002; 33 (3):361–75. Available from: https://linkinghub.elsevier.com/retrieve/pii/S0005789402800338

97. Hermida MJ, Shalom DE, Segretin MS, Goldin AP, Abril MC, Lipina SJ, et al. Risks for Child Cognitive Development in Rural Contexts. Front Psychol [Internet]. 2019 Jan 9; 9(JAN):1–12. Available from: https://www.frontiersin.org/article/10.3389/fpsyg.2018.02735/full PMID: 30687186

98. Borooah VK. Social Identity and Educational Attainment: The Role of Caste and Religion in Explaining Differences between Children in India. J Dev Stud [Internet]. 2012 Jul; 48(7):887–903. Available from: http://www.tandfonline.com/doi/abs/10.1080/00220388.2011.621945

99. UNESCO. Holistic Early Childhood Development Index (HECDI) Framework: A Technical Guide. 2014.

100. Miller AC, Murray MB, Thomson DR, Arbour MC. How consistent are associations between stunting and child development? Evidence from a meta-analysis of associations between stunting and multidimensional child development in fifteen low- and middle-income countries. Public Health Nutr [Internet]. 2016 Jun 10; 19(08):1339–47. Available from: http://www.journals.cambridge.org/abstract_S136898001500227X PMID: 26355426

101. Jeong J, Charles D, Fink G. Early Childhood Research Quarterly Pathways between paternal and maternal education, caregivers ' support for learning, and early child development in 44 low- and middle-income countries. Early Child Res Q [Internet]. 2017; 41(July):136–48. Available from: http://dx.doi.org/10.1016/j.ecresq.2017.07.001

102. Sk R, Banerjee A. Measuring the child's home learning environment and its associated factors in Malda: A micro-level study in India. Child Youth Serv Rev [Internet]. 2021 Jun; 125(October 2020):105984. Available from: https://doi.org/10.1016/j.childyouth.2021.105984

103. Rao N, Kaul V. India's integrated child development services scheme: challenges for scaling up. Child Care Health Dev [Internet]. 2018 Jan; 44(1):31–40. Available from: http://doi.wiley.com/10.1111/cch.12531 PMID: 29235171

104. Narayan J, John D, Ramadas N. Malnutrition in India: status and government initiatives. J Public Health Policy [Internet]. 2019 Mar 23; 40(1):126–41. Available from: https://doi.org/10.1057/s41271-018-0149-5 PMID: 30353132

105. Ministry of Women and Child Development. The government's efforts to fight malnutrition [Internet]. 2013. http://iap.healthphone.org/pdf/the-governments-efforts-to-fight-malnutrition.pdf

106. Nunkoo R, Ramkissoon H. Structural equation modelling and regression analysis in tourism research. Curr Issues Tour [Internet]. 2012 Nov; 15(8):777–802. Available from: http://www.tandfonline.com/doi/abs/10.1080/13683500.2011.641947

107. Bollen K. Structural Equations with Latent Variables. Wiley; 1989.

108. Hoyel RH (Ed.). Structural equation modeling: Concepts, issues, and applications. Sage Publications, Inc.; 1995.

109. StataCorp. Stata Statistical Software: Release 13. Stata: Release 13. College Station, TX; 2013.

110. Fan Y, Chen J, Shirkey G, John R, Wu SR, Park H, et al. Applications of structural equation modeling (SEM) in ecological studies: an updated review. Ecol Process [Internet]. 2016 Dec 22; 5(1):19. Available from: http://dx.doi.org/10.1186/s13717-016-0063-3

111. Sk R. Does delivery in private hospitals contribute largely to Caesarean Section births? A path analysis using generalised structural equation modelling. Joe W, editor. PLoS One [Internet]. 2020 Oct 8; 15(10):e0239649. Available from: https://dx.plos.org/10.1371/journal.pone.0239649 PMID: 33031397

112. Snopkowski K, Sear R. Does grandparental help mediate the relationship between kin presence and fertility? Demogr Res [Internet]. 2016 Mar 10; 34(1):467–98. Available from: http://www.demographic-research.org/volumes/vol34/17/

113. Hoyle R. Structural Equation Modeling for Social and Personality Psychology. Structural Equation Modeling for Social and Personality Psychology. London, England: Sage; 2011.

114. Kline RB. Principles and Practice of Structural Equation Modeling. 4th ed. Methodology in the social sciences. New York: The Guilford Press; 2015. 1–554 p.

115. Manu A, Ewerling F, Barros AJ, Victora CG. Association between availability of children's book and the literacy-numeracy skills of children aged 36 to 59 months: secondary analysis of the UNICEF Multiple-Indicator Cluster Surveys covering 35 countries. J Glob Health [Internet]. 2019 Jun; 9(1). Available from: http://jogh.org/documents/issue201901/jogh-09-010403.pdf PMID: 30410746

116. Ministry of Women and Child Development. INTEGRATED CHILD DEVELOPMENT SERVICES (ICDS) SCHEME [Internet]. 2019 [cited 2018 May 8]. https://icds-wcd.nic.in/icds.aspx
